# Intra- and Intercellular Quality Control Mechanisms of Mitochondria

**DOI:** 10.3390/cells7010001

**Published:** 2017-12-26

**Authors:** Yoshimitsu Kiriyama, Hiromi Nochi

**Affiliations:** Kagawa School of Pharmaceutical Sciences, Tokushima Bunri University, Shido 1314-1, Sanuki, Kagawa 769-2193, Japan; kiriyamay@kph.bunri-u.ac.jp

**Keywords:** mitochondria, mitophagy, transmitophgy, PGC1, mitofusin, DRP1, MIRO1, CD38

## Abstract

Mitochondria function to generate ATP and also play important roles in cellular homeostasis, signaling, apoptosis, autophagy, and metabolism. The loss of mitochondrial function results in cell death and various types of diseases. Therefore, quality control of mitochondria via intra- and intercellular pathways is crucial. Intracellular quality control consists of biogenesis, fusion and fission, and degradation of mitochondria in the cell, whereas intercellular quality control involves tunneling nanotubes and extracellular vesicles. In this review, we outline the current knowledge on the intra- and intercellular quality control mechanisms of mitochondria.

## 1. Introduction

**Figure 1:**
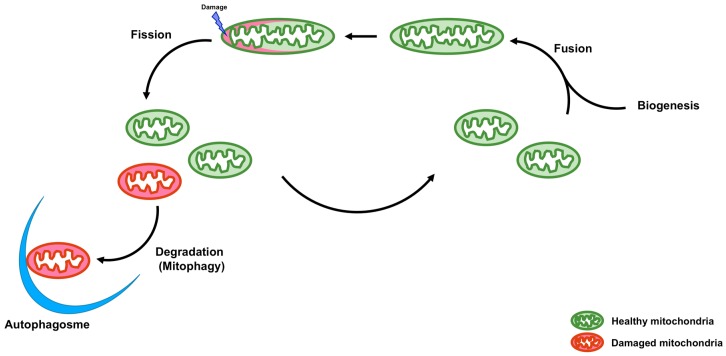
Schematic representation of intracellular quality control of mitochondria. Intracellular quality control of mitochondria consists of biogenesis, fusion, fission, and degradation (mitophagy) to maintain functions of mitochondria. Mitochondrial fission is necessary for the dissociation of damaged and dysfunctional mitochondria. Damaged and dysfunctional mitochondria are degraded by mitophagy. Mitochondrial biogenesis supplements decreased mitochondrial mass. Mitochondrial fusion leads to the exchange of mitochondrial DNA (mtDNA), proteins, and metabolites between healthy and damaged mitochondria to prevent the accumulation of damaged contents in a single mitochondrion.

**Figure 2:**
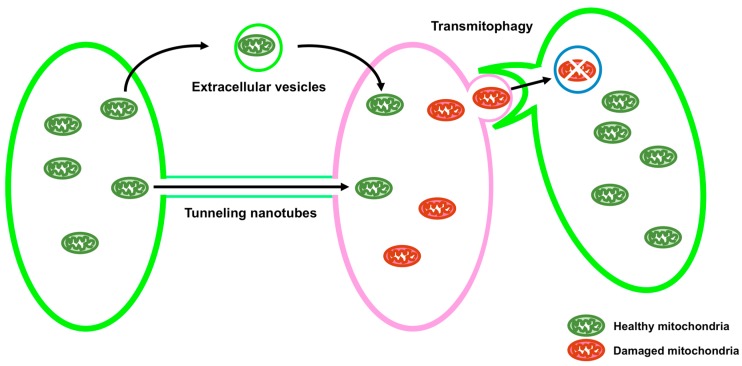
Schematic showing intercellular quality control of mitochondria. Mitochondria are transported between cells via tunneling nanotubes (TNTs) and extracellular vesicles (EVs), which help rescue cells containing damaged mitochondria by transporting healthy mitochondria from the adjacent cells. In neurons, damaged mitochondria packed in a neuron are degraded by an adjacent astrocyte (transmitophagy).

Mitochondria are double membrane organelles and are referred to as powerhouses of the cell because their major function is the generation of cellular ATP. Mitochondria also play important roles in other processes, including calcium homeostasis, reactive oxygen species production, apoptosis, autophagy, and the metabolism of amino acids, lipids, and glucose [1]. Loss of mitochondrial function is associated with various types of diseases [2]. Quality control of mitochondria is, therefore, crucial. A functional population of mitochondria is strictly controlled by intra- and intercellular quality control mechanisms. Intracellular quality control consists of biogenesis, fusion and fission, and degradation of mitochondria (Figure 1). On the other hand, intercellular quality control consists of tunneling nanotubes (TNTs) and extracellular vesicles (EVs) (Figure 2).

## 2. Intracellular Quality Control

### 2.1. Biogenesis

Mitochondria do not originate de novo; rather, proteins involved in the maintenance of mitochondrial population and mass regulate the biogenesis of mitochondria. These proteins are encoded by both mitochondrial DNA (mtDNA) and nuclear DNA (nDNA). Mitochondrial transcription factor A (TFAM), transcription factor B2, mitochondrial (TFB2M), nuclear respiratory factor 1 (NRF1) and NRF2, estrogen-related receptors (ERRs), and peroxisome proliferator-activated receptor gamma coactivator 1α (PGC-1α) play important roles in activating the transcription of genes required for mitochondrial biogenesis.

TFAM is encoded by nDNA and transported from the cytosol to mitochondria. In mitochondria, TFAM plays dual roles: as a transcription factor (TF) for mitochondrial genes and as a regulator of mtDNA replication [3,4,5]. TFB2M is also encoded by nDNA and transported from the cytosol to mitochondria to function as TF for mitochondrial genes. By contrast, transcription factor B1, mitochondrial (TFB1M), a paralog of TFB2M, is a dimethyltransferase that methylates adenine residues of mt12S rRNA [6]. TFAM binds to mtDNA and changes its structure. TFB2M and mitochondrial RNA polymerase (POLRMT) interact with TFAM to induce target gene expression. Mitochondrial transcription machinery, composed of TFAM, TFB2M, and POLRMT, initiates the expression of mtDNA [7,8,9]. A recent study has shown that POLRMT also functions as a switch between the transcription and replication of mtDNA [10]. 

NRF1 and NRF2 are nuclear TFs. NRF1 binds to GC-rich palindromes [11,12], whereas NRF2 binds to direct tandem repeats with GGAA core motifs [13,14]. Human NRF2 consists of two subunits: α and β (β1 or β2) or γ (γ1 or γ2). Subunit α contains the DNA-binding domain, whereas subunit β or γ contains the transcription activation domain [15]. Both NRF1 and NRF2 positively regulate the expression of genes encoding proteins related to the oxidative phosphorylation system (OXPHOS) complexes, heme biosynthesis, mitochondrial protein import and assembly, and mitochondrial translation [16]. NRF1 and NRF2 also regulate the expression of TFAM [17] and TFB2M [18]. 

ERRs α, β, and γ are nuclear receptors (NRs) whose endogenous ligands are unknown [19]. ERRs bind to the ERR response element with a consensus DNA sequence of TCAAGGTCA. ERRα and ERRγ bind to promoters of genes encoding mitochondrial proteins. However, the role of ERRβ in the expression of mitochondrial genes is unknown. The transcriptional function of ERRs is positively regulated by PGC-1α [20,21]. 

PGC-1α is a coactivator that lacks DNA-binding activity, but activates transcription of TFs or NRs by binding to these proteins. PGC-1α interacts with NRF1, NRF2, and ERRs to positively regulate these TFs and NRs for mitochondrial biogenesis [22]. Although PGC-1α is not a coactivator of TFAM and TFB2M, it indirectly activates the expression of TFAM and TFB2M via the activation of NRFs [17,18]. Thus, PGC-1α is considered a master regulator of mitochondrial biogenesis. PGC-1α is a member of the PGC1 family, which also includes PGC-1β and PGC-related coactivator (PRC). PGC-1β associates with NRF1 and ERRs, and positively regulates the expression of mitochondrial biogenesis proteins [22]. PRC also binds to NRF1 and ERRα. In addition, PRC forms a complex with NRF2 by binding to the host cell factor 1 (HCF-1) [23]. Post-translational modifications of PGC-1α include phosphorylation, methylation, acetylation, and deacetylation, whereas those of PGC-1β and PRC remain unclear [24].

### 2.2. Fusion and Fission

#### 2.2.1. Fusion of Mitochondria

Mitochondrial fusion results in the exchange of mtDNA, proteins, and metabolites from healthy and damaged mitochondria, in order to repress the accumulation of damaged contents in a single mitochondrion. The fusion of the outer mitochondrial membrane (OMM) is controlled by mitofusin (MFN), whereas that of the inner mitochondrial membrane (IMM) is regulated by optic atrophy 1 (OPA1). 

Two isoforms of MFN, MFN1 and MFN2, exist in mammals. Both MFNs are transmembrane GTPases, located in the OMM. MFNs play a crucial role in the tethering and fusion of the OMMs [25]. However, the GTPase and tethering activities of MFN1 are greater than that those of MFN2 [26]. Thus, MFN1 is considered the main GTP-dependent membrane tethering protein for mitochondrial fusion. MFN2 is expressed on the mitochondria-associated endoplasmic reticulum (ER) membrane. It tethers mitochondria to the ER by binding to MFNs on the OMM and functions in the uptake of mitochondrial Ca^2+^ from the ER [27]. 

OPA1 is a GTPase and transmembrane protein localized to the IMM. The long form of OPA1 (L-OPA1) is processed to its short form (S-OPA1) by OMA1 and YME1L1 [28,29]. The transmembrane domain of L-OPA1 is anchored to the IMM. By contrast, S-OPA1 is cleaved and does not possess a transmembrane domain. Knockdown of OPA1 results in mitochondrial fragmentation, which is recovered by the induction of L-OPA1 [29]. In addition, a decrease in the mitochondrial membrane potential induces complete conversion of L-OPA1 to S-OPA1 by OMA1, leading to the inhibition of mitochondrial fusion and induction of mitochondrial fragmentation [30]. Thus, L-OPA1 functions to regulate the fusion of the IMM.

#### 2.2.2. Fission of Mitochondria

It is known that mitochondrial fission is necessary for the transmission of mitochondria to daughter cells during mitosis and the dissociation of damaged DNA, proteins, and metabolites of mitochondria. Mitochondrial fission is mainly regulated by dynamin-related protein 1 (DRP1). DRP1 is a cytosolic dynamin-like GTPase. It is recruited to the OMM to form multimeric ring-like structures at mitochondrial fission sites, which leads to the constriction and scission of mitochondria in a GTPase dependent manner. The activity of DRP1 is regulated by post-translational modifications, such as phosphorylation, ubiquitination, sumorylation, *S*-nitrosylation, and *O*-GlcNAcylation [31]. 

The recruitment of DRP1 onto the OMM is mediated by OMM-localized DRP1 receptors, namely mitochondrial fission factor (MFF), fission mitochondrial 1 (FIS1), mitochondrial elongation factor 1 [MIEF1; also known as the mitochondrial dynamic 51 kDa protein (MiD51)], and MIEF2 (also known as MiD49). MFF is localized to the OMM. Overexpression of MFF leads to the recruitment of DRP1 to the OMM, whereas knockdown of MFF results in the elongation of mitochondria [32,33]. FIS1 is also an anchored membrane protein and an ortholog of yeast Fis1. Yeast Fis1 acts as a receptor for Dnm1, the yeast ortholog of DRP1, and functions in the fission of yeast mitochondria [34]. Overexpression of FIS1 enhances the activity of mitochondrial fission, whereas blocking of FIS1 results in the elongation of mitochondria [35,36,37,38]. MIEF1 and MIEF2 are anchored to the OMM. Overexpression of the MIEF proteins results in the elongation of mitochondria, due to blocking of mitochondrial fission [35,39,40]. However, depletion of MIEFs also results in the elongation of mitochondria [35,39,41]. Recently, MIEFs and MFF have been shown to coordinately function with DRP1 on the OMM and have been used as models to demonstrate the contradictory results obtained from the overexpression and knockout of MIEFs. Although both MIEFs and MFF bind to DRP1, MIEFs have higher binding affinities to DRP1 than MFF. Thus, MIEF overexpression inhibits the binding of endogenous MFF to DRP1, leading to mitochondrial fusion. MIEFs also bind to MFF, thereby linking DRP1 to MFF and forming a DRP1-MIEF-MFF complex, resulting in mitochondrial fission. MIEF knockout inhibits the formation of this complex, thus leading to mitochondrial fusion [42]. 

### 2.3. Degradation (Mitophagy)

Damaged and dysfunctional mitochondria are deleterious to the cell. Degradation of such mitochondria is, therefore, crucial. Mitochondrial degradation is executed via the process of autophagy, which removes unwanted cytosolic components [43,44,45]. Selective degradation of mitochondria via autophagy is called mitophagy. Several proteins mediate the process of mitophagy, including phosphatase and tensin homolog (PTEN), induced putative kinase 1 (PINK1), Parkin, BCL2 interacting protein 3 (BNIP3), NIX [also known as BNIP3 like (BNIP3L)], Bcl2-like protein 13 (Bcl2-L-13), and FUN14 domain containing 1 (FUNDC1).

PINK1 is a serine/threonine kinase localized to mitochondria [46]. In healthy mitochondria, presenilin-associated rhomboid-like (PARL) processes PINK1, leading to the degradation of PINK1. In depolarized mitochondria, the processing of PINK1 by PARL is blocked. This results in the accumulation of PINK1 on the OMM [47,48,49]. PINK1 undergoes autophosphorylation and phosphorylates ubiquitin moieties of originally ubiquitinated OMM proteins. In addition, PINK1 phosphorylates and activates Parkin, an E3 ubiquitin ligase that adds ubiquitin molecules to originally ubiquitinated OMM proteins [50,51,52]. PINK1 also phosphorylates the ubiquitin molecules added by Parkin. The ubiquitin-binding autophagic adaptor proteins, nuclear dot protein 52 kDa (NDP52) and optineurin (OPTN), recruit microtubule-associated protein 1 light chain 3 (LC3) to the OMM proteins ubiquitinated and phosphorylated by PINK1 and Parkin, leading to mitophagy [53,54,55]. 

BNIP3, NIX, Bcl2-L-13, and FUNDC1 are localized to the OMM and mediate mitophagy by associating with the LC3 subfamily proteins, including LC3 alpha (LC3A), LC3 beta (LC3B), and LC3C, and the γ-aminobutyric-acid-type-A receptor-associated protein (GABARAP) subfamily proteins, including GABARAP, GABARAP-like 1 (GABARAPL1), and GABARAP-like 2 (GABARAPL2) [56]. BNIP3, NIX, and Bcl2-L-13 belong to the Bcl-2 family. Although Bcl-2 harbors four Bcl-2 homology motifs (BH1–4), BNIP3 harbors only the BH3 motif [57]. Overexpression of BNIP3 leads to the induction of mitophagy [58,59], whereas BNIP3 knockdown suppresses mitophagy [60]. Phosphorylation of Ser17 of BNIP3 is necessary for the binding of BNIP3 to LC3B, whereas phosphorylation of both Ser17 and Ser24 mediates the binding of BNIP3 to GABARAPL2 (also known as GATE-16) [61]. Like BNIP3, NIX also harbors only the BH3 motif. Deletion of NIX results in defective mitophagy [62,63]. Although NIX associates with all members of the LC3 and GABARAP subfamilies [56], phosphorylation of Ser34 and Ser35 residues of NIX enhances its ability to bind to LC3A and LC3B [64]. Bcl2-L-13 is a Bcl-2 homolog protein that also plays key roles in mitophagy and mitochondrial fragmentation. Phosphorylation at Ser272 of Bcl2-L-13 is necessary for its interaction with LC3B [65]. FUNDC1, which is also located in the OMM, functions to link mitochondria with LC3. Casein kinase 2 (CK2) and Src kinase phosphorylate Ser13 and Tyr18 residues of FUNDC1, respectively, blocking the induction of mitophagy. By contrast, dephosphorylation of Ser13 and Tyr18 residues of FUNDC1 by PGAM5, a mitochondrial phosphatase, or inhibition of CK2 and Src kinase results in the induction of mitophagy [66,67].

Mitochondrial-derived vesicles (MDVs) and mitochondrial spheroids are also important pathways for mitochondrial degradation and quality control, in addition to canonical autophagy [68]. MDVs deliver oxidized components of the mitochondria to the lysosome in response to oxidative stress. MDV formation is dependent on PINK1 and Parkin. However, key proteins controlling canonical autophagy, such as ATG5 and LC3, are not necessary for MDV formation. In addition, MDV formation and turnover precede mitophagy [69,70]. A recent study has shown that Syntaxin-17 is required for fusing the MDV and the lysosome [71]. Mitochondrial spheroids are the transformed structures of the mitochondria. They have a ring or cup-like shape and are generated in response to oxidative stress and mitochondrial damage [72,73,74]. As mitochondrial spheroids are formed independently of ATG5 or ATG7, their formation is distinct from canonical autophagy. Although mitochondrial spheroids contain lysosomal proteins, the degradation of contents via mitochondrial spheroids remains to be elucidated. MFN1 and MFN2 are required for mitochondrial spheroid formation, and Parkin inhibits mitochondrial spheroid formation by degrading MFN1 and MFN2. Thus, Parkin induces mitophagy and prevents mitochondrial spheroid formation [68,72,74].

## 3. Intercellular Transport

Mitochondria are transported between cells via TNTs and EVs (Figure 2). It has been reported that intercellular transport of mitochondria via TNTs rescues cells containing damaged mitochondria by sending healthy mitochondria from adjacent cells [75,76,77,78,79,80]. The transport of mitochondria via TNTs can be bidirectional [81]. It has been shown that mitochondrial rho GTPase 1 (MIRO1), also known as ras homolog family member T1 (RHOT1), plays an important role in the intercellular transport of mitochondria through TNTs [82]. MIRO1 is anchored to the OMM and is also related to the intracellular transport of mitochondria in the cell [83]. Knockdown of MIRO1 inhibits mitochondrial transport from mesenchymal stem cells (MSCs) to epithelial cells injured by rotenone, without reducing TNT formation, whereas overexpression of MIRO1 leads to an increase in the transport of mitochondria from MSCs to rotenone-injured epithelial cells [82]. 

EVs are divided into two types: exosomes (30–150 nm) and microvesicles (MVs) (30–1000 nm) [84]. The intercellular transport of mitochondria via EVs has been demonstrated [80,85,86]. It has been recently shown that the release of mitochondria-containing EVs from the astrocyte, mediated by CD38-cyclic ADP ribose (cADPR)-Ca^2+^ signaling, saves neurons damaged by oxygen-glucose deprivation or ischemic stroke [87]. CD38 is an ADP ribosyl cyclase that generates cADPR, leading to the release of Ca^2+^ ions [88]. Activation of CD38 and addition of cADPR enhances the release of mitochondria from astrocytes, whereas knockdown of CD38 or BAPTA-AM, an intracellular Ca^2^ chelator, reduced the release of mitochondria from astrocytes [87]. Damaged mitochondria are also transported into adjacent cells for their degradation. The retinal ganglion cells produce mitochondria-containing protrusions surrounded by vesicles. The adjacent astrocyte tears off, internalizes these protrusions, and degrades the mitochondria in these protrusions. This process is termed transmitophagy [89]. 

## 4. Concluding Remarks

Although the major function of mitochondria is the generation of energy, mitochondria are also associated with cellular homeostasis, cell signaling, metabolism, and cell death. Thus, intra- and intercellular quality controls of mitochondria are crucial and rigorously regulated. The impairment of quality control results in the accumulation of damaged mitochondria, leading to cell death and various types of diseases. Modifications of molecules and/or signaling associated with intra- and intercellular quality controls of mitochondria helps rescue cells containing damaged mitochondria. Moreover, intercellular quality control of mitochondria via EVs can lead to the possibility of the delivering of healthy mitochondria into cells containing damaged mitochondria. The elucidation of detailed mechanisms of intra- and intercellular quality controls of mitochondria will help in the development of therapeutic strategies for the management of diseases caused by defective or dysfunctional mitochondria.
